# Prevention of rheumatoid arthritis: state-of-the-art and considerations for the design of new trials

**DOI:** 10.1016/j.ero.2026.100236

**Published:** 2026-09-09

**Authors:** Annette H M van der Helm - van Mil

**Affiliations:** 1 Department of Rheumatology, Leiden University Medical Centre, Leiden, The Netherlands; 2 Department of Rheumatology, Erasmus Medical Centre, Rotterdam, The Netherlands

## Abstract

Rheumatoid arthritis (RA) is among the most common chronic autoimmune diseases. Since it is well recognised that autoimmune processes may be aberrant long before clinical arthritis first develops, a research focus has shifted to the prearthritis stages of RA, under the hypothesis that these early stages are more amenable to disease-modifying interventions. This article summarises recent results from prevention trials with long-standing follow-up and discusses considerations for designing new secondary prevention trials. One of the lessons learned is that interventions for the development of anticitrullinated protein antibodies (ACPA)-positive and ACPA-negative RA can differ and that current prevention studies are labour-intensive due to long inclusion times and long follow-up periods. Importantly, the recently published European Alliance of Associations for Rheumatology (EULAR)/American College of Rheumatology (ACR) risk stratification criteria for the development of RA are valuable for selecting a homogeneous risk population and, consequently, an efficient trial design. Further research is needed to determine the best drug and the optimal endpoint. Through continued research, we hope to be able to set up efficient prevention trials and achieve personalised prevention with the right medication for the right patient at the right time.

## INTRODUCTION

Rheumatoid arthritis (RA) is among the most common chronic autoimmune diseases. The presence of clinically apparent synovitis is critical to diagnose and classify RA. It has been proven that early interventions in RA reduce the progression of joint damage and disability. Furthermore, it is well recognised that autoimmune processes may be aberrant long before clinical arthritis first develops. This has led to a focus on the prearthritis stages of RA, under the hypothesis that these early stages are more amenable to disease-modifying interventions. This article summarises recent results from prevention trials with long-standing follow-up and discusses considerations for designing new secondary prevention trials.

## RECENT TRIAL RESULTS

When the goal is to prevent RA, long-term follow-up is of crucial importance to ensure that prevention is actually achieved. Three trials with long-term data (approximately 5 years of follow-up) have recently shown results. As shown in Table [1–2], efficacy results differed for patients with anticitrullinated protein antibodies (ACPA)-positive and ACPA-negative arthralgia at increased risk of RA, and also differed somewhat when different follow-up durations after treatment cessation were assessed. ACPA-positive developing RA is the most studied. An intramuscular injection of corticosteroids, followed by 1-year of treatment with methotrexate (MTX), resulted in a short-term delay in the development of RA, a medium-term improvement in disability, symptoms, and subclinical joint inflammation (for at least 2 years), but not in sustained improvement after 5 years [1]. Abatacept resulted in a longer delay in the development of RA, lasting for several years, but here too, after approximately 5 years of follow-up, there was no difference in the incidence of RA between the treatment and placebo groups [3,4]. ACPA-negative patients with arthralgia at increased risk of RA have been included in 1 trial that showed favourable results [1]. Treatment with 1 intramuscular injection of corticosteroids plus MTX for 1 year resulted in a sustained reduction in the development of RA (number needed to treat of 4) and a durable improvement in the disease burden over the course of 5 years [1]. Although validation is needed, these data suggest that developing ACPA-positive and ACPA-negative RA should be studied separately in future trials and that tolerance is easier to induce in developing ACPA-negative RA than in ACPA-positive RA. The lessons learned to date are relevant to consider, given that conducting the TREAT EARLIER and APIPPRA trials lasted at least 10 years each [1,3].

**Table:** Overview of efficacy results of recent secondary prevention trials in reducing the development of RA and improving the disease burden

	ACPA-positive arthralgia at increased risk for RA			ACPA-negative arthralgia at increased risk for RA		
	Short-term, follow-up during treatment^a^	Intermediate including follow-up after treatment^b^	Long-term follow-up^c^	Short-term follow-up during treatment^a^	Intermediate including follow-up after treatment^b^	Long-term follow-up^c^
**Prevention of RA**						
Corticosteroids [5]	**−**	**−**	?	?	?	?
Rituximab [6]	**+**	**−**	?	?	?	?
Hydroxychloroquine [7]	**−**	**−**	?	?	?	?
Methotrexate [1]	**+**	−	**−**	**+**	**+**	**+**
Abatacept [3,4]	**+**	**+**	**−**	?	?	?
**Improving disease burden (eg, disability and symptoms)**						
Corticosteroids [5]	?	?	?	?	?	?
Rituximab [2]	**−**	**−**	?	?	?	?
Hydroxychloroquine [7]	**−**	**−**	?	?	?	?
Methotrexate [1]	**+**	**+**	**−**	**+**	**+**	**+**
Abatacept [3,4]	**+**	**−**	**−**	?	?	?

−, no benefit; +, positive effect, benefit; ?, not studied; ACPA, anticitrullinated protein antibody; RA, rheumatoid arthritis.

a Effect observed during the treatment period

b Approximately 2 years of follow-up in total

c Approximately 5 years of follow-up in total The studies in which the results are described are referenced.

## CONSIDERATIONS FOR FUTURE TRIALS

In the study design, there are 3 important choices: the intervention, the population, and the primary endpoint.

### The right drug

Regarding intervention, the choice of the appropriate agent is challenging when aiming to intervene in developing ACPA-positive RA. Besides MTX and abatacept, Plaquenil, steroids, and a single infusion of rituximab also showed no preventive effect against RA in the long term [[5], [6], [7]]. This finding may mean that other drugs are needed for the prevention of RA than those currently used to suppress disease activity. Another explanation relates to the timing of ‘the point of no return’. It is possible that the phase of arthralgia (without/with subclinical joint inflammation) is already too late and that the reversible period occurs earlier. In this context, it is interesting that the maturation of the autoimmune reaction already takes place 5 years before diagnosis, whereas symptoms appear on average 1 to 2 years before diagnosis [8]. Assuming that disease chronicity can still be influenced at the risk stage of arthralgia, it is hoped that ongoing omics analyses will provide insight into the mechanisms relevant to the final step in the development of RA from a subclinical joint inflammation, and that these findings will lead to the development of targeted treatments.

Alternatively, positive effects that are evident during treatment or persist for some time thereafter, even if not maintained in the long term, may be sufficient to support the use of the currently studied drugs for preventive treatment. Examples include the short- and intermediate-term benefits reported for MTX, rituximab, and abatacept (Table [[1], [3], [4], [7], [6], [5], [2]]).

### The right population

Trials in arthralgia, but also in patients with undifferentiated arthritis, have shown that accurate risk stratification is crucial and that heterogeneity in risk for RA can result in false negative trial results [9,10]. To obtain homogeneous risk groups, a European Alliance of Associations for Rheumatology/American College of Rheumatology (EULAR/ACR) task force developed accurate and validated risk stratification criteria for the development of RA [11]. While doing so, an important consideration was that the method should be applicable regardless of the method used to identify symptomatic persons at risk (clinical grounds [clinically suspect arthralgia {CSA}] or autoantibody-positive arthralgia) and regardless of the availability of imaging modalities for detecting subclinical joint inflammation. The risk stratification criteria consist of 6 clinical and serologic variables (Fig). The area under the curve (AUC) value was 0.80 for inflammatory arthritis development. The added value of imaging was studied. Ultrasound did not increase the discriminative ability, in contrast to magnetic resonance imaging (MRI). When an MRI scan was added, the AUC value was 0.87 for inflammatory arthritis development and 0.93 for RA development [10]. Thus, the criteria can be applied without imaging, but the highest accuracy is obtained when using an MRI scan to detect the extent of tenosynovitis as an additional risk factor. Although MRI is generally considered unfeasible (due to long scan times, the need for intravenous contrast agents, and associated high costs), recent developments in imaging techniques enable patient-friendly and affordable MRI scans without intravenous contrast agents and with a scan time of 2 to 5 minutes [12]. With regard to MRI scan interpretation, automated MRI scan evaluation based on deep learning has become possible and supportive [13]. Regardless of whether imaging is used, the risk stratification criteria contribute to an efficient study design by either including only patients with an increased or high risk, or excluding patients with a low predicted risk. The latter is valuable, since patients with a low risk may not benefit from preventive treatment.

**Figure:**
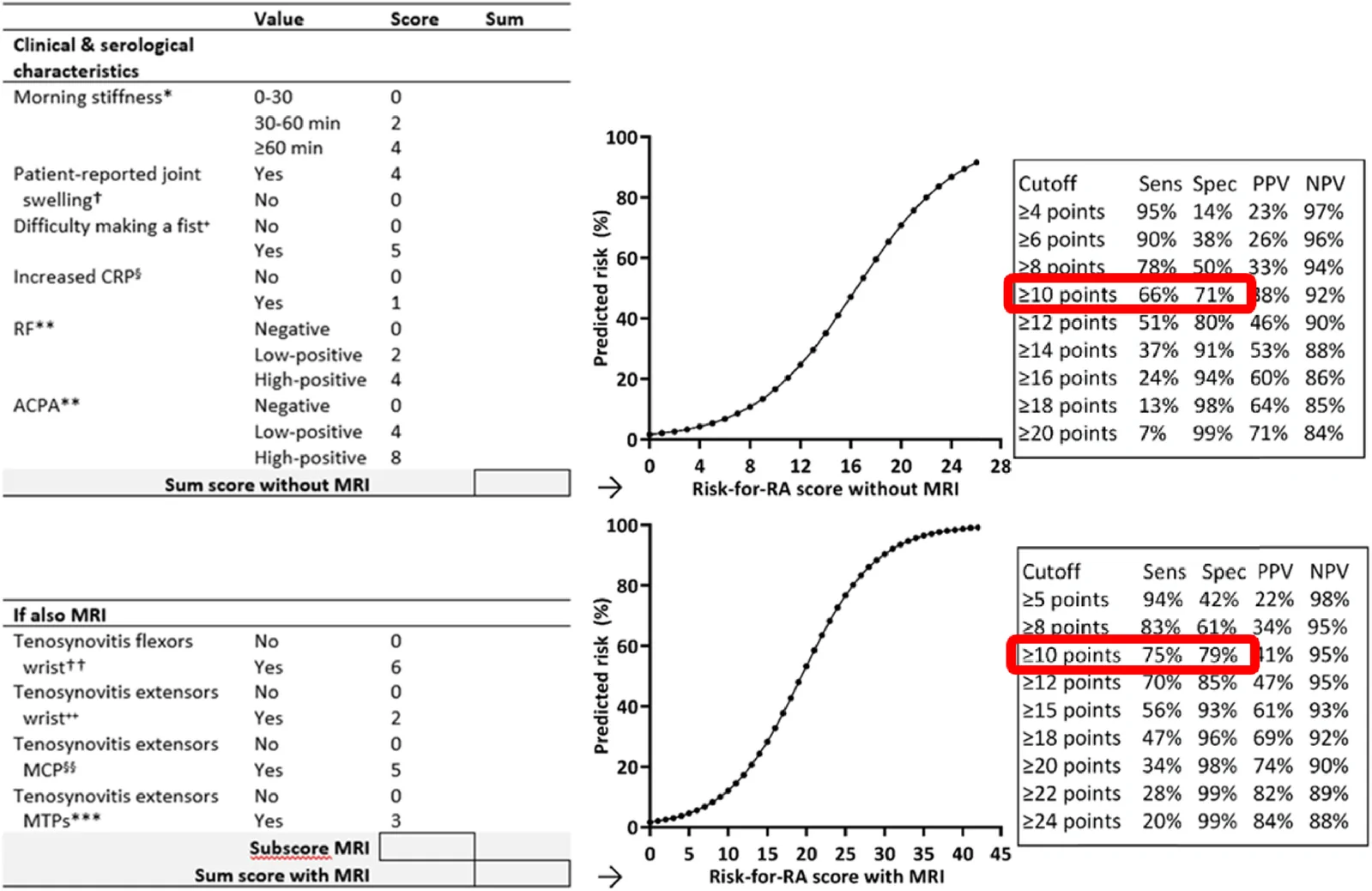
Variables included in the European Alliance of Associations for Rheumatology/American College of Rheumatology (EULAR/ACR) risk stratification criteria for rheumatoid arthritis (RA) development; for full information see van Steenbergen et al [11]. The risk stratification criteria are to be used in persons with arthralgia in secondary care without clinical arthritis, with the arthralgia not better explained by another disease/condition. The criteria can be used without or with the use of a magnetic resonance imaging (MRI) scan to depict subclinical joint inflammation. Per patient, the sum score can be obtained, corresponding risks per score can be derived from the figures in the middle column, and test characteristics per cut-off score are presented in the right column. A cut-off score of 10 had the best balance between sensitivity and specificity. For full information and explanation of variables, see van Steenbergen et al [11].

### The right primary endpoint

Regarding the choice of the endpoint: RA development as an endpoint makes a trial expensive because long-term follow-up is required. Disease burden, on the other hand, can be assessed during treatment and shortly thereafter but has also been criticised for its subjective or patient-reported nature. Therefore, it would be valuable to develop a disease activity score for CSA patients that includes a combination of (more objective and more subjective) measurements reflective of inflammation. This is an ongoing effort.

## CONCLUSION

In summary, we have learned that interventions with evolving ACPA-positive and ACPA-negative RA can differ that the EULAR/ACR risk stratification criteria are valuable for selecting a homogeneous risk group, and that further research is needed to determine the best drug and the optimal endpoint. Through continued research, we hope to be able to set up efficient prevention trials and achieve personalised prevention with the right medication for the right patient at the right time.

## CRediT authorship contribution statement

**Annette H M van der Helm - van Mil:** Writing – review & editing, Writing – original draft.
